# The adverse vascular effects of multi-walled carbon nanotubes (MWCNTs) to human vein endothelial cells (HUVECs) in vitro: role of length of MWCNTs

**DOI:** 10.1186/s12951-017-0318-x

**Published:** 2017-11-10

**Authors:** Jimin Long, Yafang Xiao, Liangliang Liu, Yi Cao

**Affiliations:** 1 Institute of Bast Fiber Crops, Chinese Academy of Agricultural Sciences, Changsha, 410205 People’s Republic of China; 2 Key Laboratory of Environment-Friendly Chemistry and Application of Ministry of Education, Lab of Biochemistry, College of Chemistry, Xiangtan University, Xiangtan, 411105 People’s Republic of China; 3 Institute of Functional Nano & Soft Materials (FUNSOM) and Jiangsu Key Laboratory for Carbon-Based Functional Materials & Devices, Soochow University, Suzhou, 215123 Jiangsu People’s Republic of China

**Keywords:** Human umbilical vein endothelial cell (HUVEC), Multi-walled carbon nanobute (MWCNT), Oxidative stress, Vascular effect, Endoplasmic reticulum (ER) stress

## Abstract

**Background:**

Increasing evidences indicate that exposure to multi-walled carbon nanotubes (MWCNTs) could induce adverse vascular effects, but the role of length of MWCNTs in determining the toxic effects is less studied. This study investigated the adverse effects of two well-characterized MWCNTs to human umbilical vein endothelial cells (HUVECs).

**Methods:**

The internalization and localization of MWCNTs in HUVECs were examined by using transmission electron microscopy (TEM). The cytotoxicity of MWCNTs to HUVECs was assessed by water soluble tetrazolium-8 (WST-8), lactate dehydrogenase (LDH) and neutral red uptake assays. Oxidative stress was indicated by the measurement of intracellular glutathione (GSH) and reactive oxygen species (ROS). ELISA was used to determine the release of inflammatory cytokines. THP-1 monocyte adhesion to HUVECs was also measured. To indicate the activation of endoplasmic reticulum (ER) stress, the expression of *ddit3* and *xbp*-*1s* was measured by RT-PCR, and BiP protein level was measured by Western blot.

**Results:**

Transmission electron microscopy observation indicates the internalization of MWCNTs into HUVECs, with a localization in nuclei and mitochondria. The longer MWCNTs induced a higher level of cytotoxicity to HUVECs compared with the shorter ones. Neither of MWCNTs significantly promoted intracellular ROS, but the longer MWCNTs caused a higher depletion of GSH. Exposure to both types of MWCNTs significantly promoted THP-1 adhesion to HUVECs, accompanying with a significant increase of release of interleukin-6 (IL-6) but not tumor necrosis factor α (TNFα), soluble ICAM-1 (sICAM-1) or soluble VCAM-1 (sVCAM-1). Moreover, THP-1 adhesion and release of IL-6 and sVCAM-1 induced by the longer MWCNTs were significantly higher compared with the responses induced by the shorter ones. The biomarker of ER stress, *ddit3* expression, but not *xbp*-*1s* expression or BiP protein level, was significantly induced by the exposure of longer MWCNTs.

**Conclusions:**

Combined, these results indicated length dependent toxic effects of MWCNTs to HUVECs in vitro, which might be associated with oxidative stress and activation of ER stress.

**Electronic supplementary material:**

The online version of this article (10.1186/s12951-017-0318-x) contains supplementary material, which is available to authorized users.

## Background

Multi-walled carbon nanotubes (MWCNTs) are among the most popular carbonaceous nanoparticles (NPs) with great uses not only in microelectronics and energy storage, but also biotechnology [1]. In biomedicine, MWCNTs have been shown great potential with many important applications, such as drug delivery and biomedical imaging [2, 3]. These biomedical applications could increase the contact of human blood vessels to MWCNTs. Therefore, it is necessary to assess the potential toxicity of MWCNTs to vascular systems in order to understand the potential adverse effects of MWCNTs entering circulation as well as to ensure the safe use of MWCNTs in nanomedicine [4, 5]. Indeed, convincing data indicated that exposure to carbonaceous NPs, including MWCNTs, could induce adverse health effects to vascular system both in vivo and in vitro [6–8], but the mechanisms remain unclear.

Multi-walled carbon nanotubes are high aspect ratio materials characterized with a high length to width ratio. Increasing evidences have shown that their potential toxicity is correlated with the physicochemical properties, e.g., length, composition and surface chemistry [9, 10]. Particularly, the length of carbon nanotubes (CNTs) has been shown to be crucial to influence their toxic potential. For example, convincing data showed that longer CNTs were more potent to induce inflammatory responses and thus promote the development of pulmonary fibrosis, which is likely associated with frustrated phagocytosis induced by longer fibers [11, 12]. Similarly, Kim et al. [13] found that longer MWCNTs were more cytotoxic to Chinese hamster ovary cells, but neither longer MWCNTs nor shorter MWCNTs induced genotoxicity in vivo or in vitro. In contrast, Han et al. [14] showed that the shorter MWCNTs were more toxic to C6 rat glioma cells, which was associated with an increase of oxidative stress. Combined, previous studies showed a crucial role of length of CNTs in determining the toxicity, which could be dependent on the endpoints evaluated and the models used. However, the association between length of MWCNTs and vascular effects is less studied at present. Cao et al. [15] showed that exposure to longer MWCNTs was associated with a stronger response in endothelial activation in vitro and plaque progression in vivo, but the longer and shorter MWCNTs used in that study had indeed similar characteristics. Donkor and Tang et al. [16] showed length dependent uptake and retention of MWCNTs in endothelial cells, but the toxicity of different MWCNTs was not further assessed in that study.

Thus, in this study we evaluated the toxicity of MWCNTs with different lengths to human umbilical vein endothelial cells (HUVECs). These MWCNTs were well-characterized that they are shorter (code XFM22) and longer (code XFM19) MWCNTs. HUVECs were selected as the in vitro model because they are among the most popular models used to evaluate the bio-effects of NPs to endothelium [4]. The cytotoxicity, oxidative stress, release of cytokines and monocyte adhesion induced by the two types of MWCNTs was compared. These endpoints were selected because they were considered as the key events associated with early development of atherosclerosis [4, 8]. To investigate the possible mechanism, the activation of endoplasmic reticulum (ER) stress biomarkers, namely the expression of *ddit3* (DNA damage-inducible transcript 3; also known as *chop*, C/EBP homologous protein) and *xbp*-*1s* (spliced X-box binding protein 1) as well as the protein level of BiP (binding immunoglobulin protein; also know as GRP78, 78 kDa glucose-regulated protein), were determined by real-time RT-PCR and Western blot, respectively. The activation of ER stress was evaluated because it has been suggested to be involved in endothelial activation [17, 18], and some recent studies indicated that NP exposure might promote dysfunction of endothelial cells through the activation of ER stress pathway [4].

## Methods

### Cell culture

Human umbilical vein endothelial cell (passage 1; purchased from ScienCell Research Laboratories, Carlsbad, CA) were cultured in supplemented endothelial medium and used at passage 3–6 as we described earlier [19]. THP-1 monocytes (ATCC) were cultured in supplemented RPMI 1640 medium (Gibco, USA) and used within 2 months as we described elsewhere [20].

### MWCNT characterization and exposure

The shorter MWCNTs (code XFM22) and longer MWCNTs (code XFM19) were purchased from Nanjing XFNANO Materials Tech Co., Ltd. The information about the physicochemical properties of XFM22 and XFM19 provided by the supplier is summarized in Table 1. For some of the experiments (see below), conductive carbon black (code XFI15; size 30–45 nm, specific surface area 120–130 mg^2^/g; purchased from Nanjing XFNANO Materials Tech Co., Ltd) was also used for comparison. In this study, the physicochemical properties of XFM22 and XFM19 were further characterized by using Raman spectra, transmission electron microscopy (TEM), BET surface area and dynamic light scattering (DLS). Raman spectra were recorded by using an inVia confocal Raman microscope (Renishaw, New Mills, Gloucestershire, UK). The morphology and structure of XFM22 and XFM19 were investigated by using TEM (FEI TECNAI G20, Hillsboro, OR, USA) accelerated at 200 kV. The TEM sizes (length and diameter) of XFM2 and XFM19 were determined by using ImagJ (NIH) based on the measurement of 20 CNTs. The specific surface area was measured by using TriStarII3020 (Micromeritics Corporate, Norcross, GA, USA). To make the suspensions of XFM22 and XFM19, a stock solution of 1.28 mg/mL particles in MilliQ water containing 2% FBS was prepared by continuous sonicating for two times of 8 min with cooling on ice using an ultrasonic processor FS-250 N (20% amplitude; Shanghai Shengxi, Shanghai, China). The stock solution was then diluted in cell culture medium to desired concentrations for exposure. To measure the hydrodynamic size and Zeta potential distribution, 16 μg/mL particles suspended in MilliQ water were prepared and analyzed by using Zetasizer nano ZS90 (Malvern, Amesbury, UK).

**Table 1:** The physicochemical properties of XFM22 and XFM19 (supplier information)

Code	Purity (%)	Diameter	Length (μm)	Special surface area (m^2^/g)	Density	Electric conductivity (s/cm)
XFM22	> 95	Outer 20–30 nm; inner 5–10 nm	0.5–2	> 110	Tap density 0.28 g/cm^3^; true density: ~ 2.1 g/cm^3^	> 100
XFM19	> 95	Outer 20–30 nm; inner 5–10 nm	10–30	> 110	Tap density 0.28 g/cm^3^; true density: ~ 2.1 g/cm^3^	> 100

### Transmission electron microscopy (TEM)

For ultrastructural observations, cells were seeded at 5 × 10^5^ on 60 mm diameter cell culture Petri dishes and grown for 2 days before exposure. The cells were exposed to 32 μg/mL XFM22 or XFM19 for 24 h, rinsed, and then scratched by using a cell scraper. After centrifuge, the cells were fixed with 2.5% glutaraldehyde in PBS overnight, post-fixed with 1% OsO_4_ for 3 h, dehydrated in a graded series of ethanol, and embedded in epoxy Resin (Epon 812). The samples were then sectioned using an ultramicrotome at 70 nm, placed on carbon film supported by copper grids, stained with uranyl acetate and lead citrate, and observed under a TEM (JEM-1230, JEOL Ltd., Tokyo, Japan) operated at 80 kV.

### Cytotoxicity

The cytotoxicity of various concentrations of MWCNTs to HUVECs was measured by water soluble tetrazolium-8 (WST-8), lactate dehydrogenase (LDH) and neutral red uptake assays using commercial kits (Beyotime, Nantong, China). WST-8 assay could reflect the viability of mitochondria in living cells because it could be converted to water-soluble yellow formazan by mitochondria. LDH could be used to indicate the integrity of membrane. And neutral red uptake assay could be used to indicate the integrity of lysosomes as it could accumulate in intact lysosomes. For the assays, 4 × 10^4^/well HUVECs were seeded on 24-well plates and grown for 2 days before exposure. Cells were then incubated with 0 μg/mL (control), 2, 4, 8, 16 and 32 μg/mL XFM22 or XFM19 for 24 h, and the cytotoxicity assays were done according to manufacturer’s instructions (Beyotime, Nantong, China). The products were read by an ELISA reader (Synergy HT, BioTek, Woburn, MA, USA). The images of HUVECs after 0 μg/mL (control), 8 and 32 μg/mL XFM22 or XFM19 exposure and neutral red staining were also taken by a light microscope (Olympus, Japan) to indicate morphological changes. For comparison, HUVECs were also exposed to various concentrations of XFI15 for 24 h, followed by WST-8 and neutral red uptake assays to indicate cytotoxicity.

### Oxidative stress

The oxidative stress in HUVECs after exposure to various concentrations of MWCNTs was indicated by the measurement of intracellular glutathione (GSH) and ROS. The intracellular GSH was measured by using a fluorescence probe monochlorobimane (MCB; Sigma-Aldrich). MCB can enter the cells freely and form a fluorescent GSH-MCB adduct catalyzed by GSH S-transferase [21]. The intracellular ROS was measured by using DCFH-DA, which can form a fluorescent product upon its reaction with a variety of ROS inside the cells [22]. Both of the assays were done as previously described [21, 22]. Briefly, 1 × 10^4^/well HUVECs were seeded on 96-well black plates and grown for 2 days prior to exposure to various concentrations of MWCNTs. After that, the cells were rinsed once, and then incubated with 50 μM MCB or 10 μM DCFH-DA in serum-free medium for 30 min. After rinsed once again, the fluorescence was read at Ex 360 ± 44 nm and Em 460 ± 40 nm (for GSH) or Ex 485 ± 20 nm and Em 528 ± 20 nm (for ROS) by an ELISA reader (Synergy HT, BioTek, Woburn, MA, USA). For comparison, HUVECs were also exposed to various concentrations of XFI15 for 24 h, and intracellular GSH and ROS were determined as indicated above.

### ELISA

The supernatants from WST-8 or neutral red uptake assays were collected and stored at − 20 °C within 1 month before analysis. The release of tumor necrosis factor α (TNFα), interleukin-6 (IL-6), soluble intercellular cell adhesion molecule-1 (sICAM-1) and soluble vascular cell adhesion molecule 1 (sVCAM-1) was determined by an ELISA kit according to manufacturer’s instruction (Neobioscience Technology Co., Ltd., China). The detection limits are TNFα 7.8 pg/mL, IL-6 3.9 pg/mL, sICAM-1 0.24 pg/mL and sVCAM-1 15.6 pg/mL. The concentrations of cytokines in all the samples are higher than the detection limits. All of the products were read by using an ELISA reader (Synergy HT, BioTek, Woburn, MA, USA), and the concentrations of each cytokine were plotted according to the standard curve.

### THP-1 adhesion

The adhesion of THP-1 monocytes to HUVECs was done as previously described [23]. Briefly, HUVECs on 96-well black plates were exposed to various concentrations of MWCNTs for 24 h. To indicate the possible role of ER stress, HUVECs were also co-exposed to an ER stress inducer thapsigargin (TG; Sigma-Aldrich, USA) before adhesion assay. TG was used as 1 μM because it has been shown before that TG at this concentration could activate ER stress pathway in HUVECs [24]. THP-1 monocytes were labeled with 10 μM CellTracker™ Green CMFDA (5-chloromethylfluorescein diacetate, Invitrogen, Carlsbad, CA), and 5 × 10^4^/well labeled THP-1 cells were incubated with the exposed HUVECs for another 1 h for adhesion. After that, the unbound THP-1 cells were washed away, and the green fluorescence from the adherent THP-1 cells was read at Ex 485 ± 20 nm and Em 528 ± 20 nm by an ELISA reader (Synergy HT, BioTek, Woburn, MA, USA).

### Western blot

The protein level of BiP (GRP78) was determined by Western blot, using α-Tubulin as internal control. 2 × 10^5^/well HUVECs were seeded in 6-well plates and grown for 2 days before exposure to 0 μg/mL (control) and 32 μg/mL XFM22 or XFM19 for 24 h. After exposure, the cells were rinsed once by Hanks solution and proteins were extracted by using cell lysis buffer for Western and IP (Beyotime, Nantong, China) following instructions. A total of 20 μL/well protein was then dissolved in NuPAGE™ 4–12% Bis–Tris protein gels and transferred to a nitrocellulose membrane (0.2 µm pore size) using iBlot^®^ 2 Gel Transfer Device (Thermo-Fisher, USA). The membrane was blocked by QuickBlock™ Blocking Buffer for Western Blot (Beyotime, Nantong, China), and then incubated with 1:1000 diluted first antibodies at 4 °C. The first antibodies are BiP rabbit monoclonal antibody (Cell Signalling Technology, USA) and α-Tubulin rabbit polyclonal antibody (Beyotime, Nantong, China). After that, the membrane was washed three times with Western wash buffer, followed by the incubation with 1:2000 diluted secondary antibody [HRP-labeled Goat Anti-Rabbit IgG (H + L); Beyotime, Nantong, China]. After 1 h incubation, the membrane was washed three times with Western wash buffer, and stained by BeyoECL Plus for 2 min (Beyotime, Nantong, China). The membrane was imaged by using FluorChem FC2 (Alpha Innotech, USA), and the relative density was determined by ImageJ (NIH).

### Quantitative real-time RT-PCR

The mRNA level of *ddit3* (*chop*) and *xbp*-*1s* were determined by quantitative real-time RT-PCR, using *gapdh* as internal control. 2 × 10^5^/well HUVECs were seeded in 6-well plates and grown for 2 days before exposure to 0 μg/mL (control) and 32 μg/mL XFM22 or XFM19 for 24 h. After exposure, the cells were rinsed once by Hanks solution and total mRNA was extracted using TRI Reagent^®^ following manufacturer’s instructions (Sigma-Aldrich, USA). The cDNA was synthesized by using HiFiScript cDNA Synthesis Kit following manufacturer’s instructions (Cwbiotech, Beijing, China). The quantitative real-time PCR was done using UltraSYBR Mixture (Cwbiotech, Beijing, China) on PikoReal™ qPCR system (Thermo-Fisher, USA). The primers for each gene are as follows: *gapdh* ACAGCCTCAAGATCATCAGC (forward primer) and GGTCATGAGTCCTTCCACGAT (reverse primer), *ddit3* GGAAACAGAGTGGTCATTCCC (forward primer) and GGAAACAGAGTGGTCATTCCC (reverse primer), *xbp*-*1s* CCGCAGCAGGTGCAGG (forward primer) and GAGTCAATACCGCCAGAATCCA (reverse primer). The mRNA levels were expressed as the ratio between the mRNA level of the target genes and the internal control gene using the comparative 2^−∆Ct^ method.

### Statistics

All the data were expressed as mean ± SD (standard deviation) of means of 3–5 independent experiments for statistical analysis [25]. Two-way ANOVA followed by Tukey HSD test was used to compare the difference in R 3.3.3 (categorical factors concentrations and lengths of MWCNTs); p value < 0.05 was considered to be statistically significant.

## Results

### Characteristics of XFM22 and XFM19

**Fig. 1:**
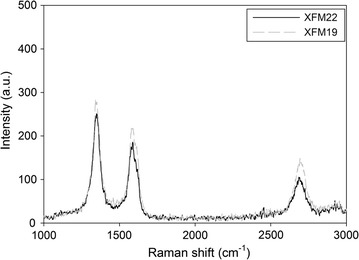
The Raman spectra XFM22 (the shorter MWCNT) and XFM19 (the longer MWCNT)

**Fig. 2:**
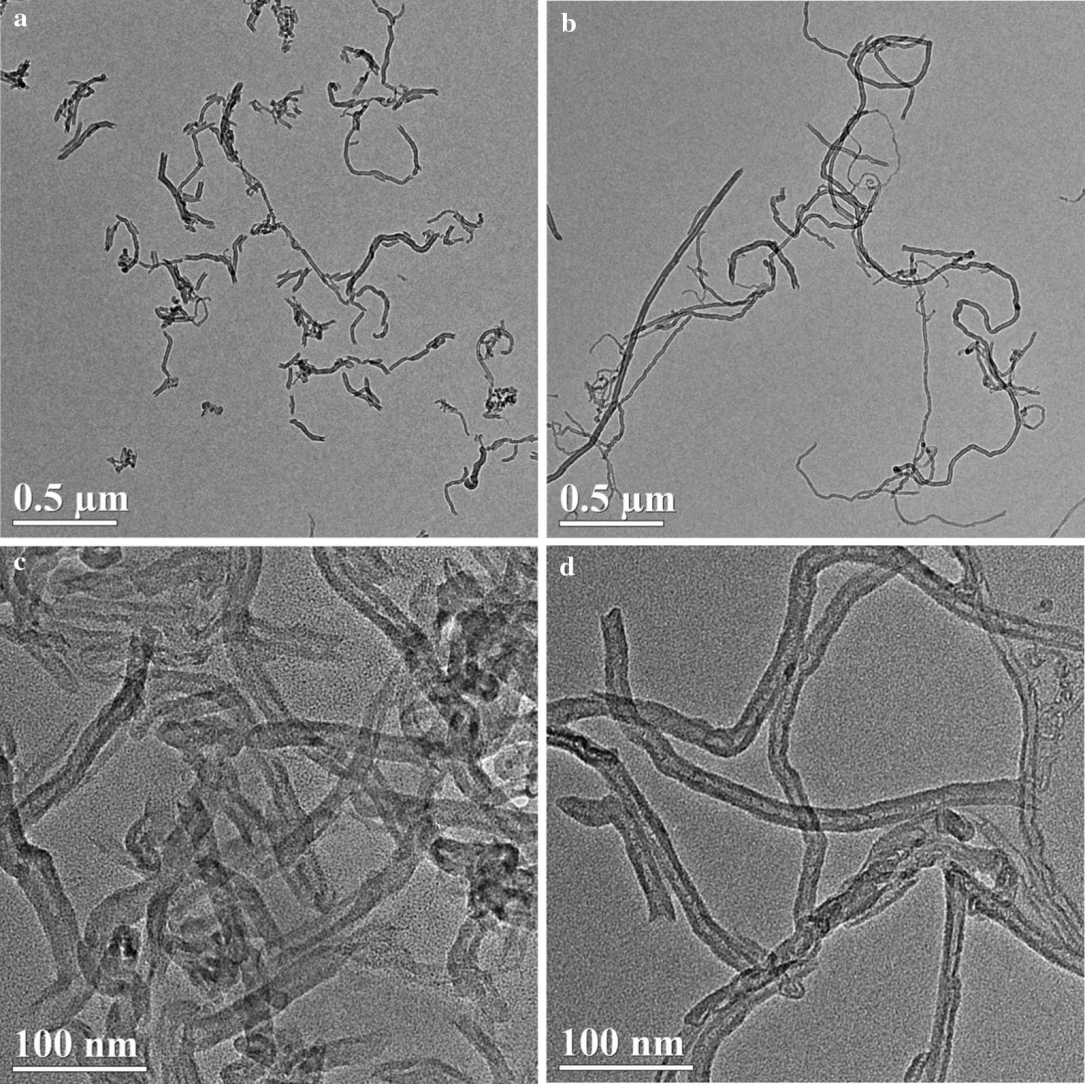
The TEM pictures of XFM22 (the shorter MWCNT; **a** and **c**) and XFM19 (the longer MWCNT; **b** and **d**) before sonication

The physicochemical properties of XFM22 and XFM19 provided by the supplier were summarized in Table 1. According to the supplier, XFM22 and XFM19 are MWCNTs with high purity and similar diameters, but the length of XFM19 is longer than that of XFM22. The Raman spectra of XFM22 and XFM19 are shown in Fig. 1, which indicates the presence of MWCNTs without impurities or surface functionalization. The TEM pictures of XFM22 and XFM19 are shown in Fig. 2, which indicates the presence of entangled and bend CNTs. The TEM pictures of sonicated XFM22 and XFM19 are shown in Additional file 1: Figure S1. No obvious change of XFM22 or XFM19 was observed before and after sonication. As summarized in Table 2, the diameters of both MWCNTs are similar, but the length of XFM19 is longer than that of XFM22. XFM22 has a relatively larger surface area and a smaller hydrodynamic size than that of XFM19. The Zeta potential of XFM 22 is almost neutral, whereas XFM19 is negatively charged. The representative hydrodynamic size and Zeta potential distribution of XFM22 and XFM19 is shown in Additional file 1: Figure S2.

**Table 2:** The physicochemical properties of XFM22 and XFM19 (measured in this study)

Code	BET surface area (m^2^/g)	TEM size	Hydrodynamic size (nm)	Zeta potential (mV)
XFM22	186.4527	Diameter 17.6 ± 5.6 nm (range 10–30 nm); length 282.1 ± 155.5 nm (range 100–600 nm)	171.4 ± 4.4	0.9 ± 0.1
XFM19	161.7611	Diameter 17.4 ± 4.6 nm (range 10–25 nm); length 883.6 ± 501.7 nm (range 200–2000 nm)	190.6 ± 2.4	− 18.9 ± 0.8

*BET* Brunauer–Emmett–Teller, *TEM* transmission electron microscope

### Internalization of MWCNTs and ultrastructural changes of HUVECs

**Fig. 3:**
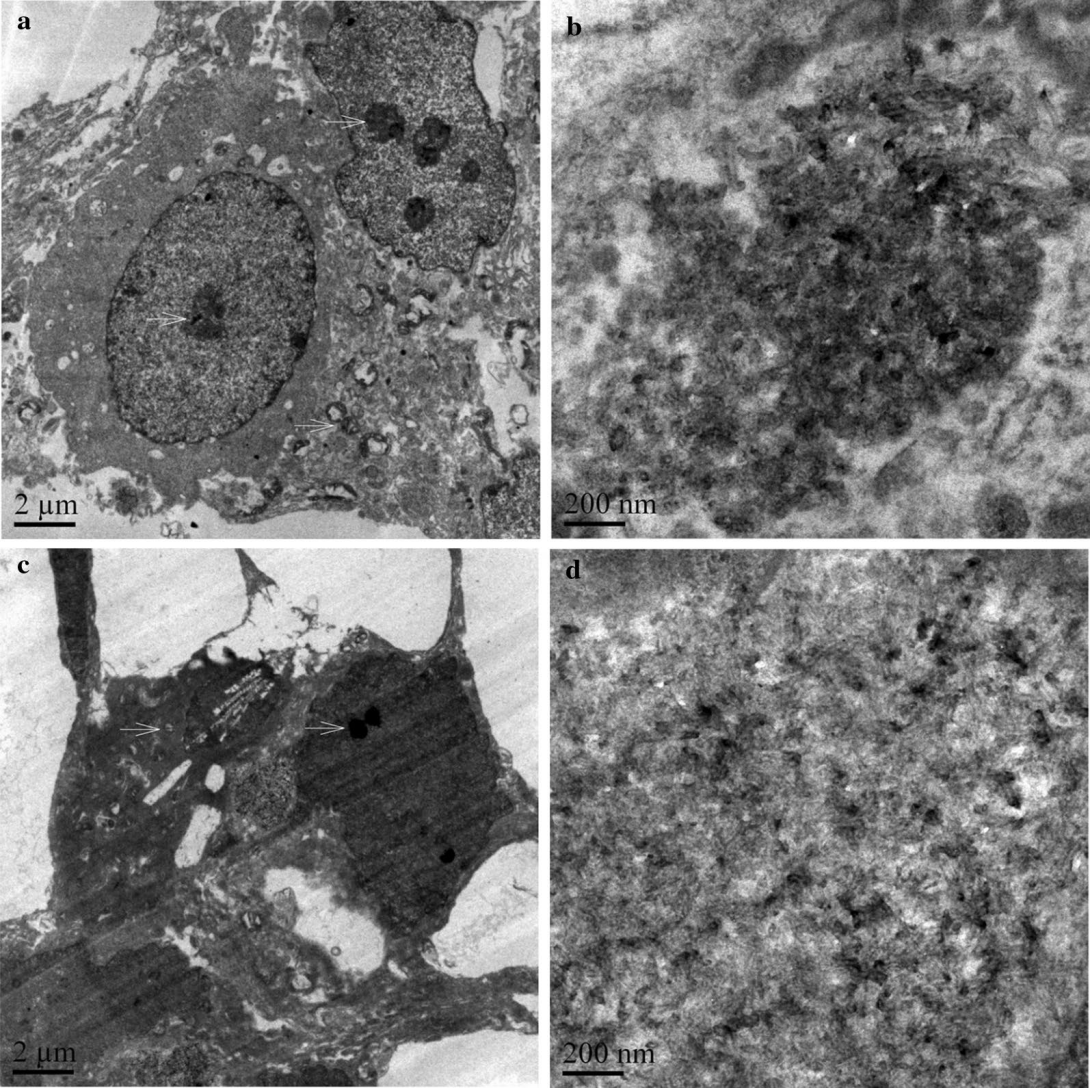
TEM images of XFM22 (the shorter MWCNT; **a** and **b**) and XFM19 (the longer MWCNT; **c** and **d**) exposed HUVECs. HUVECs were exposed to MWCNTs for 24 h, and TEM was used to indicate the internalization and localization of MWCNTs

As shown in Fig. 3, internalization of both types of MWCNTs into HUVECs was observed, with a primary localization in nuclei and mitochondria (arrows in Fig. 3a and c). In addition, there were also morphological changes of HUVECs after MWCNT exposure. While XFM22 exposed HUVECs showed normal morphologies (Fig. 3a), XFM19 exposed cells became darker with membrane damage and intracellular vacuolation (Fig. 3c).

### Cytotoxicity

**Fig. 4:**
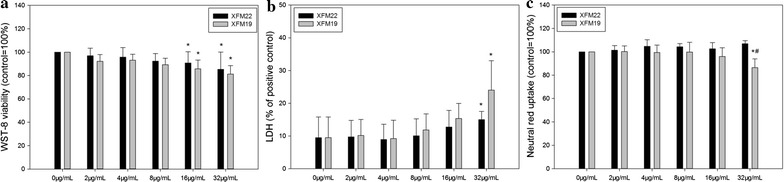
The cytotoxicity of human umbilical vein endothelial cells (HUVECs) after exposure to XFM22 (the shorter MWCNT) and XFM19 (the longer MWCNT). HUVECs were exposed to various concentrations of XFM22 and XFM19 for 24 h, and WST-8 (**a**), LDH (**b**) and neutral red uptake assay (**c**) were used to indicate the cytotoxicity of MWCNTs. *p < 0.05, compared with control; ^#^p < 0.05, comparison between XFM22 and XFM19 at the same concentration; ANOVA

The cytotoxicity of XFM22 and XFM19 was assessed by three independent assays, namely WST-8 (Fig. 4a), LDH (Fig. 4b) and neutral red uptake assay (Fig. 4c). WST-8 assay indicates a modest but significant decrease of cellular viability after exposure to 16 μg/mL (p < 0.05) or 32 μg/mL (p < 0.01) XFM22 or XFM19. For LDH assay, significantly increased LDH release was only observed after exposure to 32 μg/mL XFM22 or XFM19 (p < 0.05). For neutral red uptake assay, exposure to various concentrations of XFM22 did not significantly affect neutral red uptake (p > 0.05), whereas 32 μg/mL XFM19 significantly reduced the neutral red uptake (p < 0.05). Moreover, the neutral red uptake was significantly lower in XFM19 exposed HUVECs compared with XFM22 exposed cells (p < 0.05). The microscopic images further confirmed the loss of neutral red staining after exposure to XFM19 but not XFM22 (Additional file 1: Figure S3).

For comparison, exposure to up to 32 μg/mL XFI15 did not significantly affect cytotoxicity as assessed by WST-8 and neutral red uptake assays (p > 0.05; Additional file 1: Figure S4).

### Oxidative stress

**Fig. 5:**
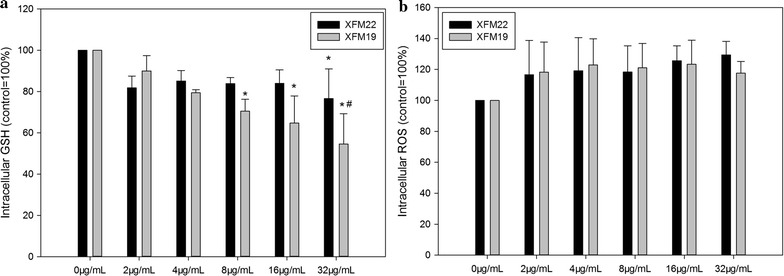
Oxidative stress in human umbilical vein endothelial cells (HUVECs) after exposure to XFM22 (the shorter MWCNT) and XFM19 (the longer MWCNT). HUVECs were exposed to various concentrations of XFM22 and XFM19 for 24 h, and intracellular GSH (**a**) and ROS (**b**) were measured to indicate oxidative stress. *p < 0.05, compared with control; ^#^p < 0.05, comparison between XFM22 and XFM19 at the same concentration; ANOVA

Oxidative stress was indicated by the depletion of intracellular GSH and increase of intracellular ROS. As shown in Fig. 5a, exposure to 8 μg/mL (p < 0.05), 16 μg/mL (p < 0.01) and 32 μg/mL (p < 0.01) XFM19 was associated with significantly decreased intracellular GSH, whereas XFM22 only significantly decreased intracellular GSH at 32 μg/mL (p < 0.05). Furthermore, the intracellular GSH concentration was significantly lower in XFM19 exposed cells compared with XFM22 exposed cells (p < 0.05). For intracellular ROS (Fig. 5b), all the concentrations of XFM22 or XFM19 only induced an insignificant increase of ROS (p > 0.05).

For comparison, exposure to various concentrations of XFI15 did not significantly affect intracellular GSH or ROS (p > 0.05; Additional file 1: Figure S5).

### Inflammatory responses

**Fig. 6:**
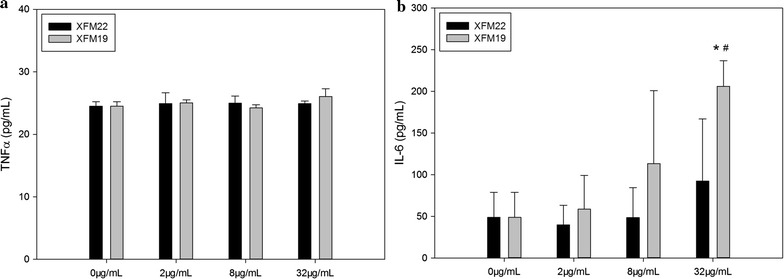
The release of inflammatory cytokines in human umbilical vein endothelial cells (HUVECs) after exposure to XFM22 (the shorter MWCNT) and XFM19 (the longer MWCNT). HUVECs were exposed to various concentrations of XFM22 and XFM19 for 24 h, and the release of TNFα (**a**) and IL-6 (**b**) was measured by ELISA to indicate inflammatory response. *p < 0.05, compared with control; ^#^p < 0.05, comparison between XFM22 and XFM19 at the same concentration; ANOVA

As shown in Fig. 6a, the release of TNFα was not significantly changed after exposure to various concentrations of XFM22 or XFM19 (p > 0.05). In contrast, exposure to 32 μg/mL XFM19 significantly increased the release of IL-6 (p < 0.05), which was significantly higher than that induced by 32 μg/mL XFM22 exposure (p < 0.05; Fig. 6b).

**Fig. 7:**
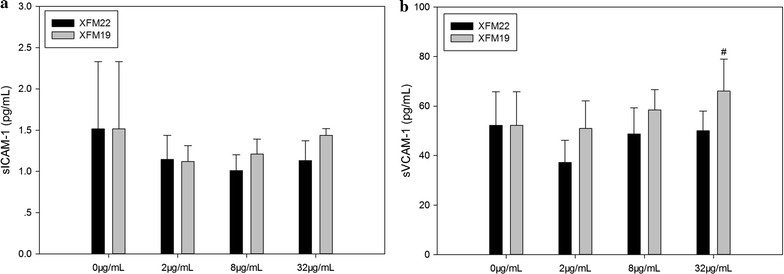
The release of soluble adhesion molecules in human umbilical vein endothelial cells (HUVECs) after exposure to XFM22 (the shorter MWCNT) and XFM19 (the longer MWCNT). HUVECs were exposed to various concentrations of XFM22 and XFM19 for 24 h, and the release of sICAM-1 (**a**) and sVCAM-1 (**b**) were measured by ELISA to indicate endothelial activation. ^#^p < 0.05, comparison between XFM22 and XFM19 at the same concentration; ANOVA

For the release of soluble adhesion molecules, exposure to various concentrations of XFM22 or XFM19 did not significantly affect the release of sICAM-1 (p > 0.05; Fig. 7a) or sVCAM-1 (p > 0.05; Fig. 7b). However, the release of sVCAM-1 induced by XFM19 exposure was significantly higher than that induced by XFM22 exposure (p < 0.05).

### THP-1 adhesion

**Fig. 8:**
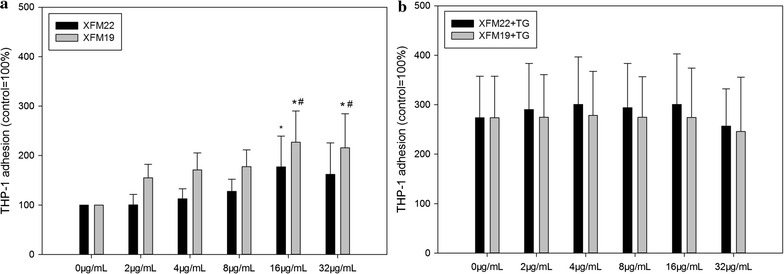
The adhesion of THP-1 monocytes to human umbilical vein endothelial cells (HUVECs) that have been exposed to XFM22 (the shorter MWCNT) and XFM19 (the longer MWCNT). HUVECs were exposed to various concentrations of XFM22 and XFM19 for 24 h (**a**), and THP-1 monocyte adhesion to HUVECs was determined by using a fluorescent probe. To induce an ER stress like condition, an ER stress inducer thapsigargin (TG) was used to co-exposure HUVECs before adhesion assay (**b**). *p < 0.01, compared with control; ^#^p < 0.05, comparison between XFM22 and XFM19 at the same concentration; ANOVA

The adhesion of THP-1 monocytes to HUVECs is shown in Fig. 8. Exposure to XFM22 or XFM19 was associated with dose-dependent increase of THP-1 adhesion, and significantly increased THP-1 adhesion was observed after exposure to 16 μg/mL XFM22 (p < 0.01), 16 μg/mL XFM22 (p < 0.01) or 32 μg/mL XFM19 (p < 0.01; Fig. 8a). In addition, compared with XFM22 at the same concentrations, exposure to 16 μg/mL (p < 0.05) and 32 μg/mL (p < 0.05) XFM19 was associated with a significantly higher THP-1 adhesion. Exposure to the ER stress inducer TG induced approximately three-fold increase of THP-1 adhesion over control, but co-exposure to XFM22 or XFM19 did not further promote TG induced THP-1 adhesion (Fig. 8b).

### Western blot

**Fig. 9:**
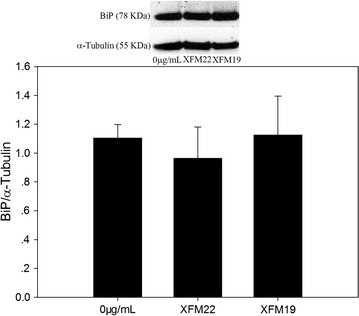
The protein level of BiP (GRP78) in human umbilical vein endothelial cells (HUVECs) after exposure to XFM22 (the shorter MWCNT) and XFM19 (the longer MWCNT). HUVECs were exposed to 0 or 32 μg/mL XFM22 and XFM19 for 24 h, and Western blot was used to determine the protein level of BiP with α-Tubulin as the internal control

The protein level of BiP, an important biomarker of ER stress, was determined by Western blot and result is shown in Fig. 9. Exposure to 32 μg/mL XFM22 or XFM19 did not significantly affect the protein level of BiP (p > 0.05).

### Real-time RT-PCR

**Fig. 10:**
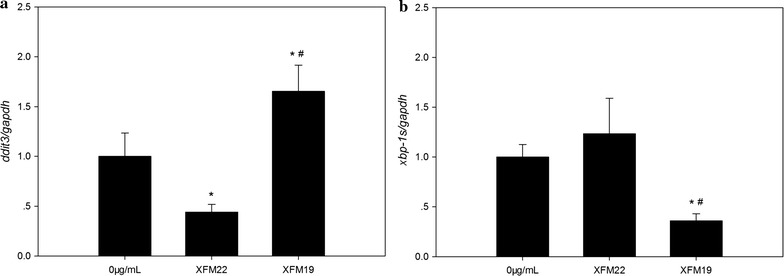
The mRNA level of *ddit3* (**a**) and *xbp*-*1s* (**b**) in human umbilical vein endothelial cells (HUVECs) after exposure to XFM22 (the shorter MWCNT) and XFM19 (the longer MWCNT). HUVECs were exposed to 0 or 32 μg/mL XFM22 and XFM19 for 24 h, and quantitative real-time RT-PCR was used to determine the mRNA level of *ddit3* and *xbp*-*1s*. The mRNA level of *gapdh* was used as the internal control. *p < 0.05, compared with control; ^#^p < 0.01, comparison between XFM22 and XFM19 at the same concentration; ANOVA

Figure 10 shows the expression of *ddit3* and *xbp*-*1s* as determined by real-time RT-PCR. Exposure to 32 μg/mL XFM19 significantly increased the expression of *ddit3* (p < 0.01), whereas exposure to 32 μg/mL XFM22 significantly decreased the expression (p < 0.01; Fig. 10a). In addition, the expression of *ddit3* in XFM19 exposed HUVECs was significantly higher than that in XFM22 exposed cells (p < 0.01). For *xbp*-*1s* (Fig. 10b), the expression was significantly decreased after XFM19 exposure (p < 0.05) but remained unaltered after XFM22 exposure (p > 0.05). The expression of *xbp*-*1s* in XFM22 exposed HUVECs was significantly higher than that in XFM19 exposed cells (p < 0.01).

## Discussion

The uses of MWCNTs in nanomedicine could increase the interactions of human blood vessels with MWCNTs, and it is necessary and urgent to evaluate the toxicity of MWCNTs to endothelial cells. More importantly, it is crucial to assess how the physicochemical properties contribute to MWCNT induced adverse effects in order to design biocompatible MWCNTs [4, 5]. In this study, we investigated the toxicity of MWCNTs of different lengths to HUVECs. The MWCNTs were well-characterized as shorter (average length about 282 nm) and longer MWCNTs (average length about 884 nm; Fig. 2 and Table 2). The shorter MWCNTs showed a larger Zeta potential compared with the longer ones, which could be due to different dispersibility of MWCNTs contributed by the lengths as suggested before [26, 27]. The results indicated that the toxicity of MWCNTs was length dependent that exposure to longer MWCNTs was associated with more morphological changes of HUVECs (Fig. 3) and relatively higher cytotoxicity to HUVECs (Fig. 4). These observations are in agreement with some previous studies showing that longer MWCNTs were more cytotoxic than the shorter ones by using different cell lines [28, 29]. However, it should be noticed that the sensitivity to MWCNTs with different length has also been suggested to be cell-type dependent [30, 31]. But we did not further try to use different types of cells to test this hypothesis. In contrast to MWCNTs, exposure to conductive carbon black was not associated with significant cytotoxicity (Additional file 1: Figure S4) or oxidative stress (Additional file 1: Figure S5), which could indicate a role of fiber-like structure in determining the toxicity of carbonaceous NPs. The longer MWCNTs (XFM19), but not the shorter ones (XFM22), significantly reduced neutral red staining in HUVECs (Fig. 4c and Additional file 1: Figure S3). Our recent studies showed that exposure to ZnO NPs significantly reduced neutral red staining without an effect on cellular viability in macrophages [20, 32]. Previous studies also showed that CNTs could accumulate into lysosomes and induce damages to lysosomes [30, 33, 34]. Therefore, the significantly reduced neutral red staining could indicate that the longer MWCNTs were more toxic to lysosomes in HUVECs.

Previous studies have shown that direct contact of engineered NPs with endothelial cells may promote endothelial activation in vitro [4, 8]. In this study, we also observed that both types of MWCNTs were capable of inducing THP-1 monocyte adhesion (Fig. 8) and release of IL-6 (Fig. 6). This is consistent with previous observations that direct exposure of endothelial cells to carbon-based NPs could induce endothelial activation [15, 35–38]. The results may also support the observations that exposure of laboratory animals to CNTs could impair the function of vascular system [15, 38–40], although the amount of translocation of CNTs into circulation remains unknown. However, in this study we did not find significantly increased release of soluble adhesion molecules after exposure to neither types of MWCNTs (Fig. 7). In our recent studies, we also found that THP-1 adhesion to HUVECs could be induced without the release of soluble adhesion molecules [41, 42]. It remains unclear if MWCNTs induced the expression of adhesion molecules without the release of soluble adhesion molecules, or if MWCNTs promoted monocyte adhesion in an adhesion molecule independent way. Interestingly, the longer MWCNTs induced a stronger response in endothelial activation in terms of THP-1 adhesion as well as release of IL-6 and sVCAM-1 (Figs. 6, 7 and 8). A previous study showed that the longer MWCNTs induced higher VCAM-1 expression in HUVECs in vitro as well as plaque progression in atherosclerotic mice in vivo [15]. However, it should be noticed that the MWCNTs used in previous study had similar characteristics, particularly when they were in suspensions [15]. Herein, by using two well-characterized MWCNTs we clearly showed that MWCNT induced endothelial activation in vitro could be length dependent.

Atherosclerosis is a disease associated with oxidative stress, and exposure to NPs has been suggested to promote endothelial activation through oxidative stress both in vivo and in vitro [6, 43]. To this end we measured intracellular GSH and ROS to indicate oxidative stress, and results showed significantly decreased intracellular GSH particularly after exposure to XFM22. However, ROS was unaltered after exposure to both types of MWCNTs (Fig. 5). Previous studies have found a crucial role of oxidative stress in MWCNT generated health effects, showing as inhibited antioxidant system (i.e., decreased antioxidant levels and inhibited antioxidant enzyme activities) and/or increased ROS [44]. Some studies also showed that the presence of ROS scavenger could alleviate MWCNT induced adverse effects to endothelial cells [35, 45], which further confirmed the role of oxidative stress. It is interesting to notice that the results from this study showed that the longer MWCNTs induced a higher depletion of intracellular GSH (Fig. 5), which may be responsible for higher toxicity of the longer MWCNTs to HUVECs.

It was recently shown that ER stress may be involved in NP-induced toxicity to endothelial cells [4]. ER is a crucial organelle involved in proper function of cells. Perturbation of normal function of ER could lead to ER stress, which has been suggested to mediate endothelial activation in metabolic diseases [17, 46]. To indicate the possible role of ER stress in MWCNT-induced toxicity to HUVECs, we measured the biomarkers of ER stress. It was shown that exposure to XFM19, but not XFM22, was associated with significantly increased expression of *ddit3* but not that of *xbp*-*1s* or BiP protein level (Figs. 8 and 9). A recent study also showed significantly increased DDIT3 protein level in mouse macrophages after exposure to anodic alumina nanotubes, a kind of engineered NPs with high aspect ratio similar to MWCNTs [47]. *ddit3* is a transcription factor that could regulate a number of inflammatory cytokines, such as IL-6 [17, 18]. In our recent study, we also showed that stressing HUVECs with ER stress inducer TG significantly promoted IL-6 release from HUVECs [48]. Thus, the fact that XFM19 provoked a stronger response in IL-6 release observed in this study could be due to the activation of *ddit3*. However, it should be noticed that compared with a previous report showing up to about 15-fold increase of *ddit3* expression in ZnO NP exposed HUVECs [49], the response of *ddit3* observed in this study was more modest. Moreover, the expression *xbp*-*1s* and BiP protein level was not significantly increased after exposure to XFM22 or XFM19 (Figs. 9 and 10). This is in contrast to previous reports showing the increase of these ER stress biomarkers in ZnO NP, Au NP and CdTe quantum dot exposed HUVECs [49–51]. Therefore, we proposed that exposure to MWCNTs might only induce a modest activation of ER stress. Co-exposure to TG did not further promote MWCNT-induced THP-1 adhesion to HUVECs (Fig. 8), which indicated that HUVECs with ER stress might not be more sensitive to MWCNT exposure.

In this study, the concentrations of MWCNTs were used from 2 to 32 μg/mL. Some studies investigated the toxicity of CNTs following intravenous administration. For example, Ma et al. [52] recently showed that intravenous injection of mice with 4 mg/kg CNTs (equals to about 50 μg/mL in blood) disrupted iron homeostasis and induced inflammation. Similarly, Zhang et al. [53] found that injection with 0.5 mg/kg MWCNTs (corresponds to 6.25 μg/mL) induced immunotoxicity in mice, but the PEGylated MWCNTs were less toxic compared with the pristine ones. In contrast, Tang et al. [54] and Ahmadi et al. [55] did not find significant toxicological responses in mice even after intravenous administration of CNTs up to 150 μg/mouse (about 75 μg/mL in blood). The different responses observed in different studies could be contributed by the physicochemical properties of CNTs used. For nanomedicinal studies, Wang et al. [56] and Kafa et al. [57] injected mice intravenously with 50 μg/mouse MWCNTs (about 25 μg/mL in blood). Both of the studies showed that MWCNTs could accumulate into brains, which indicated that they could be used for drug delivery and bio-imaging. In a different studies, Antaris et al. [58] used as low as 4 μg/mouse CNTs (about 3.2 μg/mL in blood) for dual imaging/photothermal therapy. The concentrations used in this study were within the concentrations that might happen in vivo.

In summary, the adverse effects of two well-characterized MWCNTs to HUVECs were investigated in this study, and the results indicated that the toxicity of MWCNTs was length dependent. The longer MWCNTs were more cytotoxic and promoted a stronger response in endothelial activation associated with higher depletion of intracellular GSH and *ddit3* expression, which suggested a role of oxidative stress and ER stress. The results also suggested that the shorter MWCNTs may be safer for nanomedicinal applications, and it is necessary to limit the contact of longer MWCNTs with human endothelial cells to avoid the adverse health effects.

## Additional file

**Additional file 1.** Additional Figures S1–S5.
