# Supplementary material for: The adverse vascular effects of multi-walled carbon nanotubes (MWCNTs) to human vein endothelial cells (HUVECs) in vitro: role of length of MWCNTs
Source: J Nanobiotechnology. 2017 Nov 10;15:80. doi: 10.1186/s12951-017-0318-x (PMC5681822; doi:10.1186/s12951-017-0318-x)
Supplement: Supplementary file 1 — Additional file 1. Additional Figures S1–S5. [file 12951_2017_318_MOESM1_ESM.docx]

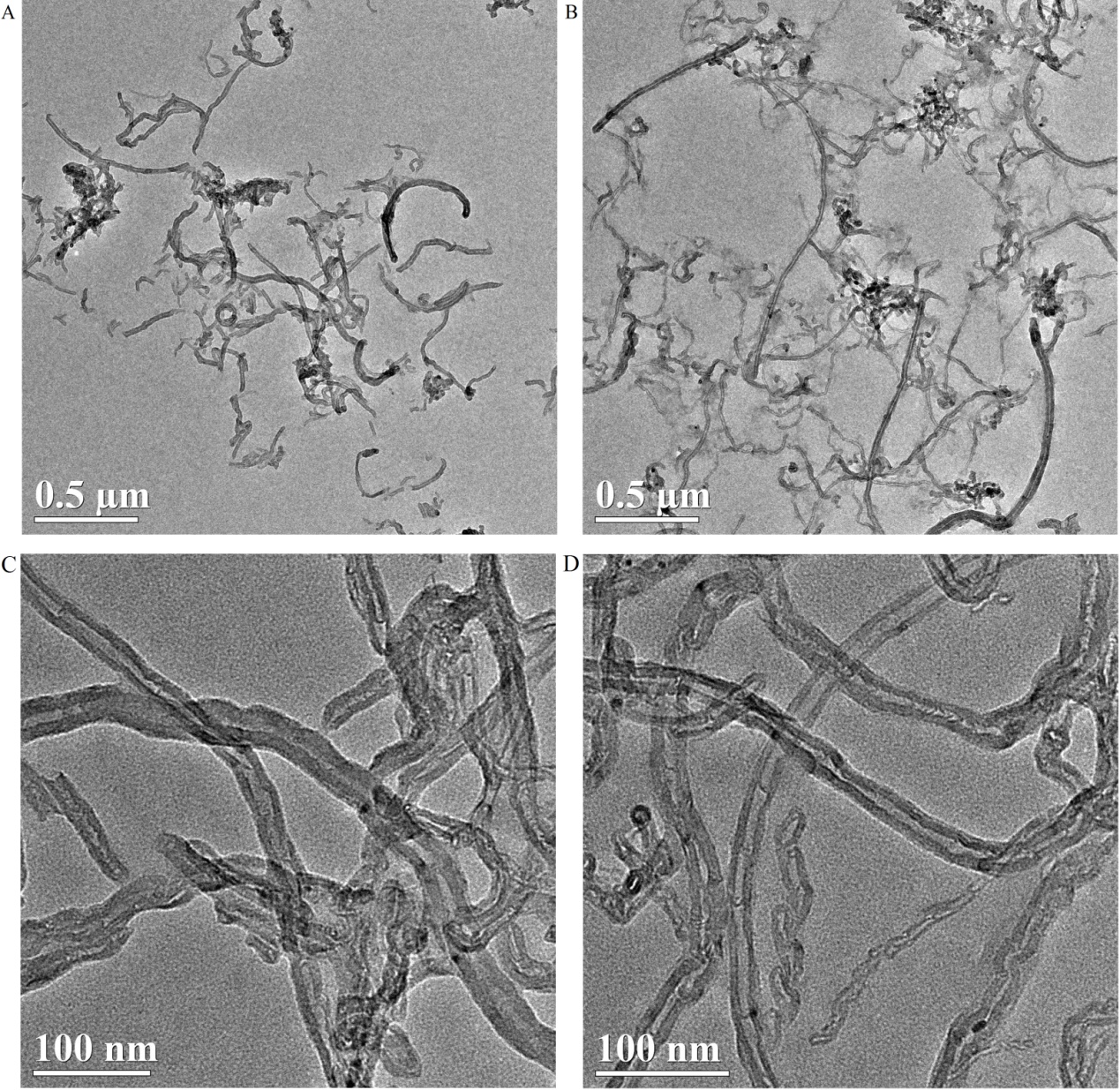


Figure S1. The TEM pictures of XFM22 (the short MWCNT; S1A & S1C) and XFM19 (the long MWCNT; S1B & S1D) after sonication.


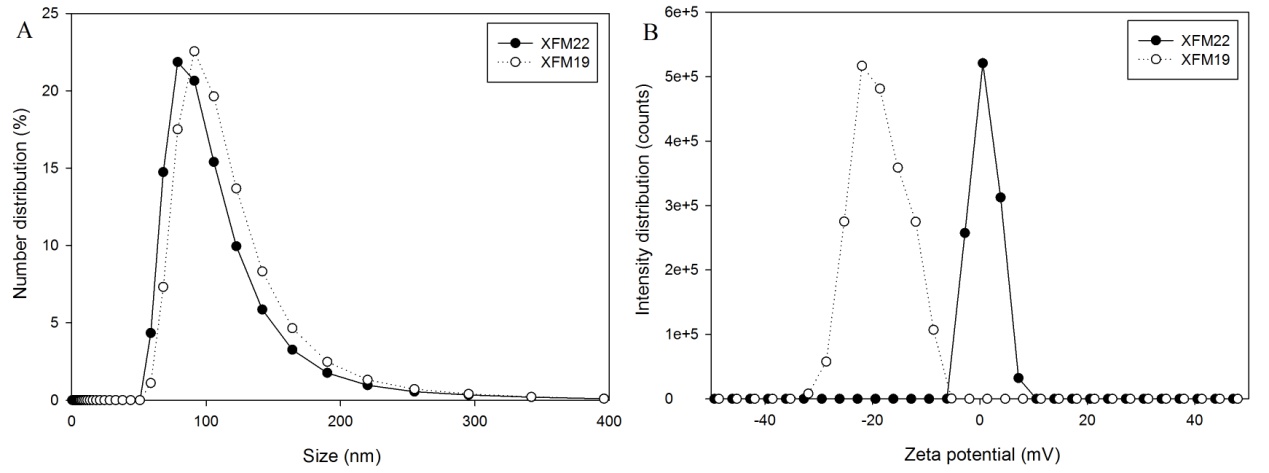


Figure S2. The hydrodynamic size (S2A) and Zeta potential (S2B) distribution of XFM22 and XFM19. Data are representatives from three measurements.


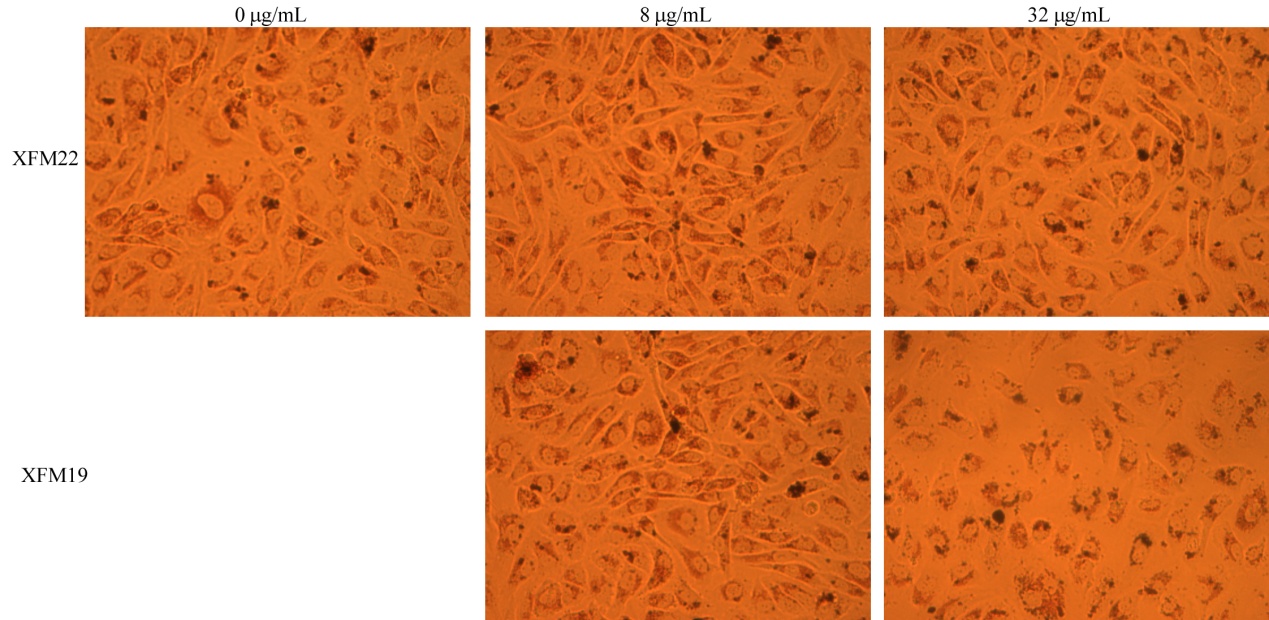


Figure S3. Representative images showing 0 μg/mL (control), 8 μg/mL and 32 μg/mL XFM22 or XFM19 treated human umbilical vein endothelial cells (HUVECs). The intact lysosomes were stained by neutral red (red color).


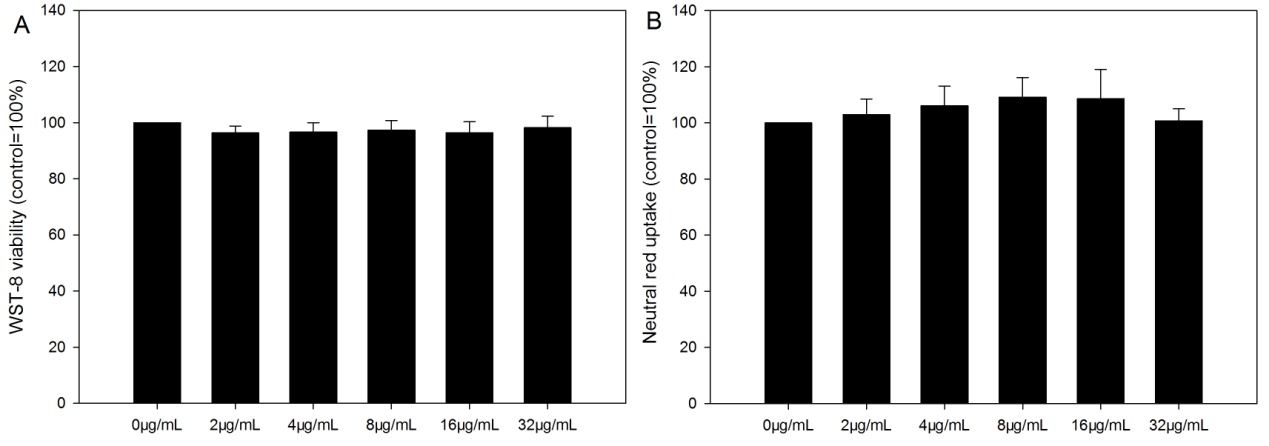


Figure S4. The cytotoxicity of human umbilical vein endothelial cells (HUVECs) after exposure to XFI15 (conductive carbon black). HUVECs were exposed to various concentrations of XFI15 for 24 h, and WST-8 (S4A) and neutral red uptake assay (S4C) were used to indicate the cytotoxicity of conductive carbon black. Data represent mean±SD of four independent experiments (n=3).


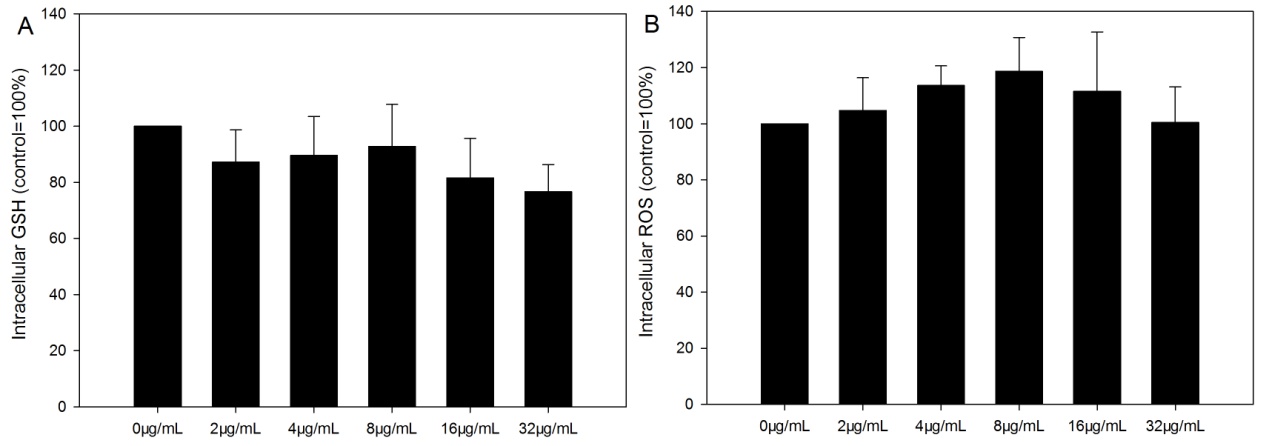


Figure S5. Oxidative stress in human umbilical vein endothelial cells (HUVECs) after exposure to XFI15 (conductive carbon black). HUVECs were exposed to various concentrations of XFI15 for 24 h, and intracellular GSH (S5A) and ROS (S5B) were measured to indicate oxidative stress. Data represent mean±SD of four independent experiments (n=3).
